# MoS_2_ Nanodonuts for High-Sensitivity Surface-Enhanced Raman Spectroscopy

**DOI:** 10.3390/bios11120477

**Published:** 2021-11-25

**Authors:** Samar Ali Ghopry, Seyed M. Sadeghi, Cindy L. Berrie, Judy Z. Wu

**Affiliations:** 1 Department of Physics and Astronomy, University of Kansas, Lawrence, KS 66045, USA; 2 Department of Physics, Jazan University, Jazan 45142, Saudi Arabia; 3 Department of Physics, The University of Alabama, Huntsville, AL 35899, USA; ss0013@uah.edu; 4 Department of Chemistry, The University of Kansas, Lawrence, KS 66045, USA; cberrie@ku.edu

**Keywords:** TMD nanodonuts, graphene, nanohybrids, biosensing, surface-enhanced Raman spectroscopy

## Abstract

Nanohybrids of graphene and two-dimensional (2D) layered transition metal dichalcogenides (TMD) nanostructures can provide a promising substrate for extraordinary surface-enhanced Raman spectroscopy (SERS) due to the combined electromagnetic enhancement on TMD nanostructures via localized surface plasmonic resonance (LSPR) and chemical enhancement on graphene. In these nanohybrid SERS substrates, the LSPR on TMD nanostructures is affected by the TMD morphology. Herein, we report the first successful growth of MoS_2_ nanodonuts (N-donuts) on graphene using a vapor transport process on graphene. Using Rhodamine 6G (R6G) as a probe, SERS spectra were compared on MoS_2_ N-donuts/graphene nanohybrids substrates. A remarkably high R6G SERS sensitivity up to 2 × 10^−12^ M has been obtained, which can be attributed to the more robust LSPR effect than in other TMD nanostructures such as nanodiscs as suggested by the finite-difference time-domain simulation. This result demonstrates that non-metallic TMD/graphene nanohybrids substrates can have SERS sensitivity up to one order of magnitude higher than that reported on the plasmonic metal nanostructures/2D materials SERS substrates, providing a promising scheme for high-sensitivity, low-cost applications for biosensing.

## 1. Introduction

Surface-enhanced Raman spectroscopy (SERS) provides an important analytical tool for biosensing with high sensitivity that depends on two mechanisms: an electromagnetic mechanism (EM) and a chemical mechanism (CM) [1,2,3,4]. The measured Raman signal intensity of a molecule is directly related to the electromagnetic field intensity that acts on the molecule. Plasmonic nanostructures can be implemented to generate a much enhanced evanescent field around the molecules through the excitation of so-called localized surface plasmon resonances (LSPR) on plasmonic nanostructures. The SERS EM enhancement factor can be up to 10^8^ and is therefore dominant in SERS enhancement [5,6,7] In addition to the SERS EM enhancement, the CM effect that stems from the charge transfer between the molecule and the substrate can add further SERS enhancement with an enhancement factor up to 10^3^ shown experimentally [8,9,10]. 2D atomic materials such as graphene and transition metal dichalcogenides (TMDs) [11,12] are excellent choices as SERS substrates due to their atomically flat sheet morphology that facilitates adsorption of probe molecules on the substrate surface and enables efficient charge transfer via weak interactions, such as van der Waals (vdW) or π-π interactions, between the probe molecules and substrates. The EM and CM enhancements could be obtained on the same SERS substrate via integration of plasmonic metal nanostructures, such as nanoparticles (NPs), nanorods, etc., with graphene or other 2D heterostructures [8,13] in nanohybrids, allowing higher SERS enhancement factors to be achieved through superposition of the EM and CM effects [14,15,16,17,18].

While metallic plasmonic nanostructures have been the major focus to induce EM enhancement, nonmetallic ones have recently emerged as a promising alternative to address the drawbacks of the metallic plasmonic nanostructures including strong spectra noise background due to contamination on the metal surface, the deformation and distortion of the probe molecules due to the strong metal-adsorbate interactions, the metal-catalyzed side reactions of the probe molecules and substantial joule dissipation [9,10,19,20]. The unique optoelectronic and photonic properties of 2D atomic layers of TMDs [21] make them promising for applications in SERS substrates for both EM and CM enhancement effects with a reduced joule dissipation anticipated on non-metallic plasmonic nanostructures [22,23,24,25]. In a recent exploration of SERS EM enhancement in nanodiscs (N-discs) of TMDs (WS_2_ and MoS_2_) of lateral dimension ~500 nm and height ~3–5 nm grown on the monolayer of graphene (TMD N-discs/graphene), [26] a strong LSPR effect was observed and attributed to photo-induced carrier doping of TMD N-discs and TMD/graphene interfacial dipole-dipole interaction. This results in a strong SERS EM enhancement with an extraordinary Rhodamine 6G (R6G) SERS sensitivity of 5 × 10^−12^ M on the TMDs N-discs/graphene nanohybrids [26]. It should be noted that the LSPR effect in TMD nanostructures could be comparable or higher than that of their metallic or other semiconductor counterparts [27,28]. Using R6G peak intensity at 613 cm^−1^ on different substrates, the EM enhancement factors could be estimated using graphene only SERS substrate as reference. Specifically, the enhancement factors of 10.3 and 8.7, respectively, are obtained by dividing the intensities of the 613 cm^−1^ peak on TMDs (WS_2_) N-discs/graphene and gold nanoparticles (AuNPs) on graphene (or AuNPs/graphene) SERS substrates by that on graphene [29]. While the values confirm both exhibit a strong LSPR effect, the LSPR of the non-metallic TMD N-discs is 40% higher than their metallic counterparts. In addition, further enhancement has been demonstrated via the superposition of the LSPR effects of the TMD N-discs and Au plasmonic nanoparticles [17,30].

Quantitatively, SERS enhancement relies on many factors such as the size and the shape of plasmonic nanostructures and substrate morphology [31,32,33,34]. For example, Hong et al. examined SERS enhancement using 4-nitrothiophenol (4-NTP) and 4-aminothiophenol (4-ATP) as probe molecules by adsorbing them on AuNPs of the average lateral size of 17, 30, 40, 50, 60, and 80 nm [29]. With increasing AuNPs lateral size, the SERS enhancement was found to increase initially until reaching the highest at ~50 nm, followed by a decrease with further AuNP size increase. The observed trend was attributed to the combined effects of AuNP size and shape for maximizing probe molecule adsorption, minimizing light scattering, and optimizing plasmonic electromagnetic field configuration. Stamplecoskie et al. examined the correlation between the SERS enhancement and AgNP lateral size using R6G as a probe [35] and observed a similar trend of SERS enhancement with AgNP size variation with the optimal SERS enhancement at AgNPs of ~50 nm in the lateral dimension. On the other hand, plasmonic nanostructures of different shapes have been fabricated including Au nanospheres [36], Au nanostars [37], Au nanorods [38], and so on [39,40]. Li et al. compared SERS enhancement on Au nanobipyramids and Au nanorods and found much stronger SERS signals in the former [41]. Fan et al. investigated SERS of graphene oxide (GO) hybrids with different Ag nanostructures including nanospheres, nanocubes, and nano-octahedral [14], and observed a strong shape effect on SERS enhancement. The highest SERS enhancement was obtained on the Ag nano-octahedra/GO nanohybrids.

In this work, we explore the growth of MoS_2_ nanodonuts (N-donuts) by controlling the size of the (NH_4_)_2_MoS_4_ precursor nuclei via variation of its concentration in the range of 0.06−0.40 wt%. The hypothesis is that MoS_2_ N-donuts could be achieved on large-size precursor nuclei due to the nonuniform conversion to MoS_2_ along the radial direction while MoS_2_ N-discs form on smaller nuclei when such nonuniformity is negligible. In addition, the solution-based deposition may lead to nonuniform distribution as the solvent is evaporated, which could also lead to donut-shaped structures. With increasing precursor concentrations, the precursor nuclei dimension increases monotonically and MoS_2_ N-donuts become predominant at precursor concentration of 0.26 wt% or higher. Interestingly, a resonant (at 532 nm excitation) R6G SERS sensitivity ~2 × 10^−12^ M has been achieved on these MoS_2_ N-donuts/graphene SERS substrates, in contrast to 5 × 10^−12^ M for the 0.06 wt% MoS_2_ where MoS_2_ N-discs are more prevalent [26]. More importantly, the obtained SERS sensitivity is more than one order of magnitude higher than that of the AuNPs/graphene and TMDs/metal nanostructure SERS substrates [42,43,44,45], indicating that the nonmetallic TMD plasmonic nanostructures can provide a promising alternative to metallic ones for SERS and other applications that require light management.

## 2. Materials and Methods

### 2.1. Growth of Graphene Using Chemical Vapor Deposition

Graphene was synthesized on polycrystalline Cu foils (Alfa Aesar, 99% purity) using chemical vapor deposition (CVD) and the details of the growth procedure have been described in detail in our earlier works [46,47,48]. Briefly, graphene was grown on the copper foil placed inside a quartz tube in the CVD furnace at 1050 °C for a growth time of about 30 min in the flow of a mixed gas of H_2_ (7 sccm) and CH_4_ (40 sccm). For graphene transfer on SiO_2/_Si substrates via a wet transfer procedure [46,47], 3% Poly (methyl methacrylate) (PMMA) was spin-coated at 3000 rpm for 30 s over the graphene/Cu sample, followed with sample baking at 120 °C for 5 min in the air on a hotplate. The Cu foil was removed by soaking the PMMA/graphene/Cu (Cu side facing down) samples in copper etchant (FeCl_3_ solution) for about 4 h, followed by rinsing the PMMA/graphene samples at least five times with deionized water. The PMMA/graphene samples were then placed onto the SiO_2/_Si and left overnight to dry at room temperature in air. Afterward, the PMMA/graphene/SiO_2_/Si stack was soaked in acetone four times to remove PMMA. Finally, the samples were rinsed with isopropanol (IPA) and thermally annealed at 400 °C for 30 min in forming gas (Ar (500 sccm)/H_2_ (300 sccm)) to remove PMMA residues on graphene.

### 2.2. Synthesis of MoS_2_ N-Donuts on Graphene

MoS_2_ was grown on graphene using a vapor transport process established in our previous works [26,49]. To obtain MoS_2_ N-donuts, the (NH_4_)_2_MoS_4_ precursor solution of variable concentrations was prepared by dissolving (NH_4_)_2_MoS_4_ powder in (N,N-dimethylformamide, DMF) with different concentrations of 0.06 wt%, 0.10 wt%, 0.20 wt%, 0.26 wt% and 0.40 wt%. It should be noted that continuous layers of MoS_2_ were achieved at ~0.10 wt% with multiple precursor dips [49]. while MoS_2_ N-donuts could be obtained on graphene with a single dip of the precursor of low (0.06 wt%) to high (0.40 wt%) precursor concentrations. However, at the lower end of precursor concentrations in this range, MoS_2_ N-discs seemed preferable due to possibly the small precursor nuclei dimension. The nuclei dimension increases with the precursor concentration, MoS_2_ N-donuts became predominant at the higher end of precursor concentrations. After the graphene/SiO_2_/Si samples were dipped in the precursor solution, spinning at 3000 rpm for 30 s was followed to achieve a uniform coating of the precursor on graphene. The samples were annealed afterward in a tube furnace at 450 °C for 30 min in sulfur vapor transported by a mixed gas of H_2_ (10 sccm) and Ar (50 sccm) from the sulfur powder placed upstream from the sample in a quartz tube at about 200 °C.

### 2.3. Characterization of MoS_2_ N-Donuts on Graphene

The sample phase and morphology were characterized using Raman spectroscopy and microscopy (WiTec alpha 300 confocal Raman system), and atomic force microscopy (AFM). For Raman characterization of graphene, 488 nm excitation laser was employed using a 20× microscope objective. The integration time was 3 s and the laser power was ~1–5 mW. For the Raman spectroscopy and maps of TMDs, a 100× microscope objective was used with either 532 or 488 nm laser excitation. For consistency, Raman maps were collected at a minimum of 4 different locations on each sample. AFM images were collected using a Digital Instruments Multimode Nanoscope IIIA AFM system in contact mode using standard silicon nitride cantilevers (k = 0.06, 0.27 N/m). Images were collected in at least three locations on the sample surface at multiple scan sizes. The average diameter, height, and density of the MoS_2_ N-donuts were measured using the Nanoscope software (Bruker, Version 1.5). Specifically, densities were measured using the particle analysis feature, and the diameters and heights were measured by taking cross-sections through at least 20 MoS_2_ N-donuts for each sample.

### 2.4. Raman Spectra of R6G on SERS Substrates

For the investigation of SERS, R6G was used as a probe molecule. A droplet of R6G (diameter around 4 mm and concentration from 5 × 10^−5^ M to 10^−13^ M) was placed on the substrate surface and left to dry for about one hour on a hotplate set at 70 °C. The diameter of the R6G samples on the substrate after it dried is around 4–5 mm. R6G SERS characterization was carried out using laser excitation of 532 nm on the same WiTec alpha 300 confocal Raman system. The laser beam spot was about tens of µm that is further reduced through a 20× microscope objective to allow multiple scan spots near the center of the droplet for consistency. An integration time of 3 s and a low intensity (~1–5 mW) were used to avoid the possible damage of the R6G probe molecules. For an improved signal-to-noise ratio, each presented Raman spectrum is an average of 5–10 spectra gathered at the same spot of the sample. It should be noticed that every Raman spectrum demonstrated in this work is representative because it is based on an average of ~10 or more Raman spectra gathered at different spots arbitrarily chosen on a sample and typically 2–3 samples were tested for reproducibility. To prevent the coffee-ring effect, the tested spots were chosen not too close to the R6G sample’s edge.

### 2.5. Finite-Difference Time-Domain Simulation

A finite-difference time-domain simulation (FDTD) study of the characteristic plasmonic effect was carried out to reveal the differences between MoS_2_ N-donuts and MoS_2_ N-discs. A commercial software package Device Multiphysics Simulation Suite of Lumerical software (2020a version) was used. Our simulation model is mostly used to highlight how these two structures support different plasmonic field enhancement factors, defined as the ratios of the field intensities in the presence of the N-donuts and N-discs to those in their absence. Simulations were carried out considering metallic N-donuts and N-discs with different sizes, placed on SiO_2_ substrates.

## 3. Results and Discussion

**Figure 1:**
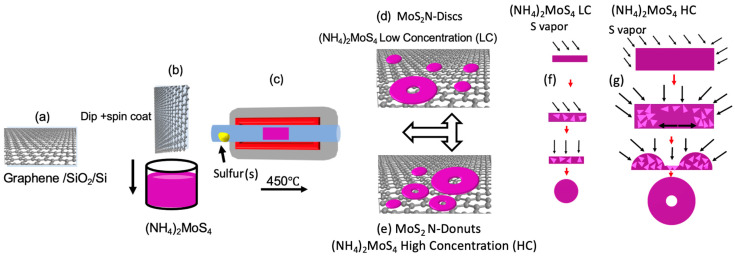
Schematic description of the synthesis process (**a**) graphene transferred on SiO_2/_Si substrates via a wet transfer procedure (**b**,**c**) MoS_2_ on graphene using the vapor transport process. (**d**) synthesis of the MoS_2_ N-Discs with low concentrations of (NH_4_)_2_MoS_4_ precursor solution and (**e**) synthesis of the MoS_2_ N-donuts with high concentrations of (NH_4_)_2_MoS_4_ precursor solution. The hypothetical growth mechanism MoS_2_ N-discs at low (**f**) and MoS_2_ N-Donuts at high (**g**) (NH_4_)_2_MoS_4_ precursor concentration, respectively. Triangles represent MoS_2_ nuclei formed during the vapor transport annealing process.

Figure 1a–g demonstrates schematically the process established in this work for synthesis of MoS_2_ N-donuts/graphene nanohybrids (see Method for details). Controlling the (NH_4_)_2_MoS_4_ precursor concentration in a DMF solution was found critical to obtaining the MoS_2_ N-donuts. N-donuts nanostructures can be obtained on graphene with precursor concentrations as low as 0.06 wt% while precursor nuclei lateral dimension increases with the precursor concentration. At lower precursor concentrations, MoS_2_ N-discs seem to be dominant on smaller precursor nuclei (Figure 1d). MoS_2_ N-donuts become dominant when larger precursor nuclei emerged at higher precursor concentrations as illustrated in Figure 1e. This means the amount of the precursor available on the sample surface affects the nucleation and evolution of the MoS_2_ nanostructures during the MoS_2_ formation. While the detailed mechanism requires further investigation, we hypothesize that the lateral and vertical dimensions of the precursor nuclei formed at the initial stage of the vapor transport process may increase monotonically with the precursor concentration. As the precursor nuclei’s lateral and vertical sizes become large enough at around 0.2 wt% or higher precursor concentrations, the conversion of the precursor to MoS_2_ during the vapor transport process may become nonuniform with the conversion occurring at the edges of the nuclei first. The converted MoS_2_ edge may facilitate the transport of the remaining precursor at the center of the nuclei towards the edge, resulting in the formation of the MoS_2_ N-donuts as depicted schematically in Figure 1f.

Figure S1a exhibits the Raman spectra taken on three samples including the pristine graphene (green, inset), MoS_2_/graphene nanohybrids made with 0.10 wt% precursor concentration (red, with MoS_2_ N-discs dominant) and with 0.26 wt% precursor concentration (blue, MoS_2_ N-donuts dominant) (Supporting Information). The graphene’s G peak at ~1600 cm^−1^ due to the primary in-plane vibration mode and 2D peak at ~2707 cm^−1^ due to secondary in-plane vibration of zone-boundary phonons [50], and D peak at ~1356 cm^−1^ (associated with the defect in graphene^8^) are clearly observable on these samples. On the pristine graphene, the intensity ratio of 2D to G peak (I_2D_/I_G_) is around 1.8 while I_D_/I_G_ is negligible, indicating that the graphene is a monolayer with high quality. On the MoS_2_/graphene nanohybrid made with 0.10 wt% precursor concentration, the graphene’s Raman peak intensities are notably enhanced most probably due to the LSPR effect by the MoS_2_ nanostructures [26]. The enhancement factor of the graphene G peak is ~5. Interestingly, a higher enhancement factor of ~8 can be observed on the graphene G peak of the MoS_2/_graphene nanohybrid sample made with 0.26 wt% precursor concentration, indicating a stronger LSPR effect. It should be noted that such an enhancement in the Raman signatures of graphene by metallic plasmonic nanostructures has been reported on metal-nanostructures/graphene nanohybrid samples [13,51,52]. A similar enhancement observed on MoS_2_/graphene nanohybrids indicates that the LSPR can be effectively generated on nonmetallic TMD nanostructures. Figure S1b displays the Raman spectra of MoS_2_ taken on the MoS_2_/graphene nanohybrids made with 0.10 wt% (red) and 0.26 wt% (blue) precursor concentrations, respectively, using Raman excitation at 488 nm. The Raman peaks in both spectra can be indexed to the $E_{2g}^{1}$ at ~393 cm^−1^ (due to S and Mo atoms in-plane displacement mode) and $A_{1g}$ at ~419 cm^−1^ (due to the S atoms out of displacement mode) of MoS_2_ [53], confirming MoS_2_ was grown on graphene in the MoS_2_/graphene nanohybrid samples.

**Figure 2:**
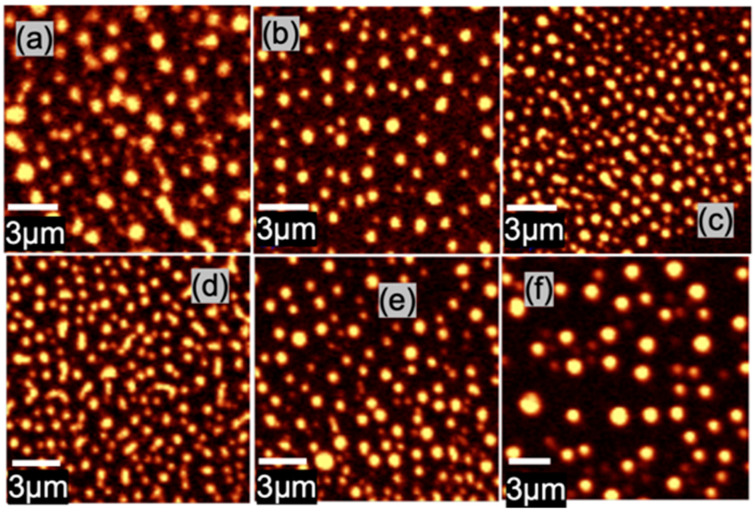
MoS_2_ A_g1_ peak Raman maps of MoS_2_ N-donuts and N-discs/graphene samples that were synthesized with different precursor solution concentrations of (**a**) 0.06 wt%, (**b**) 0.10 wt%, (**c**) 0.20 wt%, (**d**) 0.26 wt%, (**e**) % 0.32 wt%, and (**f**) % 0.40 wt%.

Figure 2a–f show Raman maps of MoS_2_ A_g1_ peak taken on the MoS_2_/graphene nanohybrid samples made with different precursor solution concentrations of (a) 0.06 wt %, (b) 0.10 wt %, (c) 0.2 wt %, (d) 0.26 wt %, (e) 0.32 wt%, and (f) 0.40 wt%. The Raman maps show that the MoS_2_ formed is not in a continuous layer, rather in the morphology of NPs of approximately round shape in the selected (NH_4_)_2_MoS_4_ precursor concentration range. Quantitatively, the (NH_4_)_2_MoS_4_ precursor concentration exhibits an effect on both the MoS_2_ NP density and shape. At the lowest precursor concentration of 0.06 wt%, the round shape of MoS_2_ NPs grows in two different sizes: smaller size with an average diameter of ~200 nm (majority) and larger size with an average diameter of ~1.0 µm with low density. With increasing precursor concentrations in the range of 0.20–0.26 wt%, the size of the MoS_2_ NPs changes moderately in the range of 300–600 nm, while the NP density increases. Above the precursor concentration of 0.26 wt%, the lateral dimension of the MoS_2_ NPs increases considerably and the NP density decreases.

**Figure 3:**
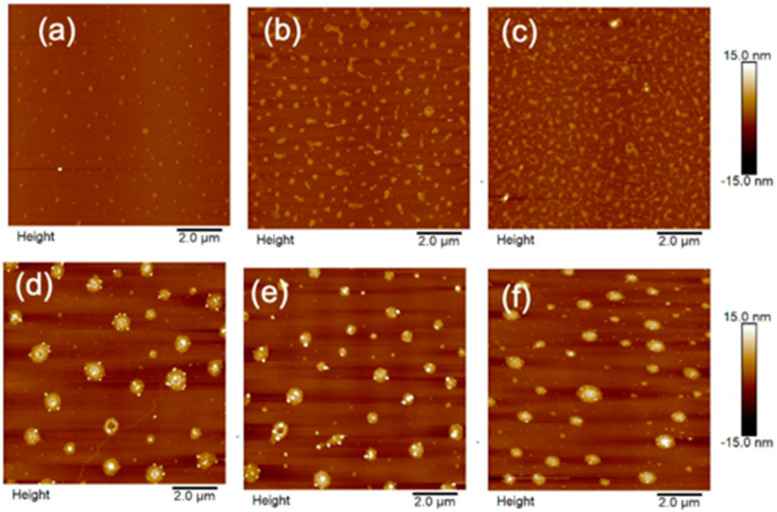
Contact mode AFM images (10 µm × 10 µm) of MoS_2_ samples that were synthesized with different precursor solution concentration. (**a**) 0.06 wt%, (**b**) 0.10 wt %, (**c**) 0.20 wt%, (**d**) 0.26 wt%, (**e**) 0.32 wt%, and (**f**) 0.40 wt%.

**Figure 4:**
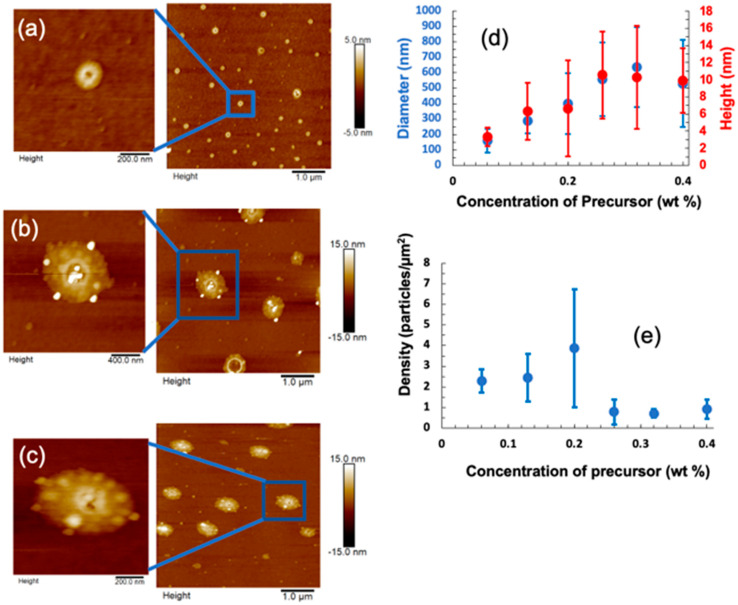
(**a**–**c**) Representative contact AFM images (5 µm × 5 µm) along with images of individual features from samples synthesized from MoS_2_ precursor concentrations of (**a**) 0.06 wt% (**b**) 0.26 wt% and (**c**) 0.40 wt% with zoom in. (**d**) Average diameter (blue) and height (red) of MoS_2_ NPs as a function of precursor concentration. (**e**) Average density of MoS_2_ NPs as a function of precursor concentration. Error bars represent the standard deviation of the measurement.

A more quantitative measurement of the morphology and density of the MoS_2_ NPs is shown in Figure 3 and Figure 4 at different precursor concentrations using AFM. The discontinuous morphologies of the MoS_2_ NPs have been confirmed in the entire range of the precursor concentration (Figure 3) and the result agrees well qualitatively with the Raman maps in Figure 2. However, the better spatial resolution in AFM images in Figure 3 suggests the MoS_2_ NPs tend to have irregular shapes at lower precursor concentrations while becoming more round-shaped at higher precursor concentrations of 0.26 wt% or higher.

AFM images of 5 µm × 5 µm at selected concentrations of 0.06 wt%, 0.26 wt%, and 0.40 wt% are shown in Figure 4a–c along with zoomed-in images of single MoS_2_ N-donuts and N-discs features. Interestingly, MoS_2_ N-discs of smaller lateral dimensions are the dominant features at lower precursor concentrations. In contrast, MoS_2_ N-donuts have a larger lateral dimension and are present even in samples made at the lowest precursor concentration of 0.06 wt%. With increasing precursor concentration, the proportion of the MoS_2_ N-donuts increases, and in the samples made with precursor concentrations of 0.26% or higher, MoS_2_ N-donuts become the dominant features. The average dimensions and density of the features are shown in Figure 4d,e as a function of the precursor concentration. At low precursor concentrations, the MoS_2_ NP diameter is between 200 to 400 nm, the height is 2–7 nm, and the density is 2–4 particles/µm^2^. Diameters and height increase up to 600 nm and 10 nm, respectively, with increasing precursor concentration which results in NP density decreasing to 1.5 to 1.0 particles/µm^2^. As shown in Figure 4d, the average diameter of the N-discs and N-donuts increases monotonically with the (NH_4_)_2_MoS_4_ precursor concentration from ~200 nm at 0.06 wt% precursor concentration to 600 nm at 0.26 wt% concentration. However, the average diameter seems to saturate at higher precursor concentrations.

**Figure 5:**
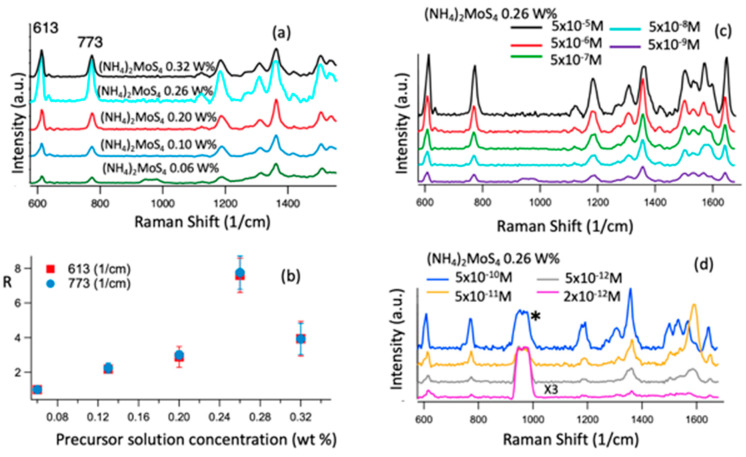
(**a**) Average Raman spectra of R6G molecules at the concentration of 5 × 10^−5^ M, collected from six batches of the samples shown in Figure S2, deposited on five MoS_2_/graphene nanohybrids substrates, MoS_2_ was synthesized with a precursor solution concentration of 0.06 wt% to 0.32 wt%. (**b**) The corresponding enhancement factor of the 613 and 773 cm^−1^ R6G peaks intensity on the five samples that were synthesized with different precursor solution concentrations with respect to the one was synthesized with a precursor solution concentration of 0.06 wt%. (**c**,**d**) Raman spectra of the R6G molecules with different concentrations in the range of 5 × 10^−5^ M to 5 × 10^−9^ M (**c**), and 5 × 10^−10^ M to 2 × 10^−12^ M (multiplied by 3) (**d**) on the MoS_2_/graphene nanohybrids substrates, MoS_2_ was synthesized with a precursor solution concentration of 0.26 wt%. The * mark denotes the Si peak.

Figure 5a displays the comparison of the Raman spectra of R6G (5 × 10^−5^ M) probe molecules on MoS_2_/graphene nanohybride substrates that were synthesized with different precursor solution concentrations from 0.06 wt% to 0.32 wt%, measured using the same excitation of 532 nm wavelength. Most of the R6G Raman peaks are visible on all spectra. Especially, the spectra include the R6G fundamental peaks at 613 cm^−1^ allocated to the C–C–C ring in-plane vibration mode, 773 cm^−1^ assigned to aromatic C–H bending mode, 1190 cm^−1^ and 1648 cm^−1^ allocated to C–O–C stretching mode and the C–C stretching mode, respectively [20,54]. Among the five spectra, the R6G spectra on the lowest precursor concentration of 0.06 wt% (green) have the lowest R6G peak intensity, indicative of the lowest SERS enhancement. It should be mentioned that MoS_2_ N-discs are predominant in this sample. In contrast, the highest SERS enhancement is observed on the spectrum for MoS_2_/graphene nanohybride made with 0.26 wt% precursor concentration (black) on which MoS_2_ N-donuts are predominant. Quantitatively, the enhancement factor can be estimated using the ratio of the intensity of the R6G Raman peaks on the MoS_2_/graphene samples made using different precursor concentrations at 613 cm^−1^ and 773 cm^−1^ to the ones on the MoS_2_ (0.06 wt%)/graphene). Figure 5b exhibits the estimated enhancement factor as a function of precursor concentration of the five samples shown in Figure 5a. The maximum enhancement factor of 8.2 was obtained on the MoS_2_/graphene sample at 0.26 wt% precursor concentration, suggesting that the SERS enhancement is a compromise of the shape, dimension, and density of MoS_2_ NPs. Interestingly, MoS_2_ N-donuts are predominant in MoS_2_/graphene nanohybrides when the precursor concentration is exceeding ~0.20 wt%. The higher SERS sensitivity in this precursor concentration range, especially the peak SERS sensitivity in MoS_2_/graphene nanohybride made from 0.26 wt% precursor concentration, suggests that MoS_2_ N-donuts may support LSPR in a more preferable way than other N-discs or other shapes of MoS_2_ nanostructures.

The R6G molecule droplets of varying concentrations ranging from 5 × 10^−5^ M to 2 × 10^−12^ M were cast and dried on the substrate and then Raman spectra were gathered with a 532 nm laser. The R6G Raman spectra are compared at the R6G concentrations of 5 × 10^−5^ M to 5 × 10^−9^ M in Figure 5c, and the R6G concentrations of 5 × 10^−10^ M to 2 × 10^−12^ M in Figure 5d, respectively. All Raman signatures peaks of R6G are obvious at R6G concentrations above 5 × 10^−10^ M. With further reduction of the R6G concentration to 2 × 10^−12^ M, most of the R6G Raman signature peaks are still detectable, particularly the vibrational modes with larger polarizability, such as 613 cm^−1^, 773 cm^−1^, 1191 cm^−1,^ and 1648 cm^−1^. Therefore, the R6G sensitivity of 2 × 10^−12^ M on the MoS_2_ N-donuts/graphene nanohybrid is about half an order of magnitude higher than that obtained on MoS_2_ N-discs/graphene [26] and about one order of magnitude higher than the best so far reported using the plasmonic metal nanostructure/graphene substrates [17].

**Figure 6:**
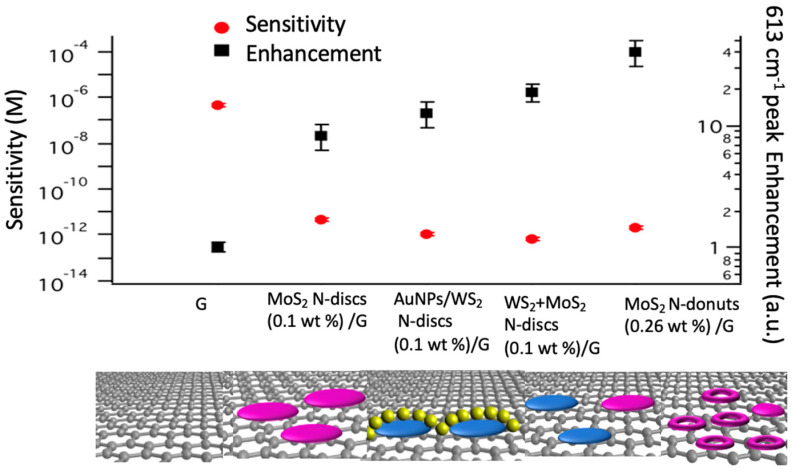
Comparison of SERS Sensitivity and R6G 613cm^−1^ peak enhancement on different SERS substrates: graphene, MoS_2_ N-discs (0.10 wt%)/graphene, AuNPs/MoS_2_ N-discs (0.10 wt%)/graphene, WS_2_ N-discs +MoS_2_ N-discs (0.10 wt%)/graphene and MoS_2_ N-donuts (0.26 wt%)/graphene.

Figure 6 shows the comparison of SERS Sensitivity and R6G 613 cm^−1^ peak enhancement factor (normalized with respect to that on graphene only SERS substrates) on different SERS substrates of graphene only, MoS_2_ N-discs/graphene [26], AuNPs+MoS_2_ N-discs (0.10 wt%)/graphene [29], WS_2_ N-discs+MoS_2_ N-discs (0.10 wt%)/graphene [55] and MoS_2_ N-donuts/graphene (made from 0.26 wt% precursor concentration in this work). The result shows that the best enhancement was obtained on the MoS_2_ N-donuts/graphene.

The R6G Raman peak intensities at 613 cm^−1^ and 773 cm^−1^ as a function of the R6G concentration are depicted in Figure S3a–d for the MoS_2_ (0.26 wt%)/graphene. Figure S3a,b are in linear scale while Figure S3c,d, in semi-logarithmic scale. The curves can be fitted approximately with an equation of y=logx+m as shown in Figure S3. Indeed, this logarithmic relation between the SERS intensity and concentration is noted in earlier reports of SERS sensitivity on a variety of analyte molecules [15,19,56,57].

The experimental results presented in this paper suggest a higher field enhancement for the case of TMD nanodonuts which may also depend on the details of the size and shape of the particles. The focus of our simulations is to examine the plasmonic features of such nanostructures and their differences with NDs. For this, we adopted a phenomenological approach that can reveal these issues without dealing with the detailed microscopic treatment of plasmonic features of the TMD NDs and nanodonuts, which is out of our current scope. For this, we chose Ag as a material model. Of course, such a material structure is very different from the MoS_2_ material studied in this paper. However, since LSPRs are ubiquitous optical signatures of charge carriers, as shown below, our model can explain the impact of the shapes of MoS_2_ structures studied in this paper on the SERS enhancement. The simulations were done considering a normal planar polarized light reaching an NDs or N-donut placed on a SiO_2_ substrate (Figure 1). The substrates were considered to be air. The mesh size in the x–y plane is considered to be 4 nm and along the z-axis 0.5 nm. The extinction spectra of the structures were obtained as 1-T, wherein T was the transmittance.

**Figure 7:**
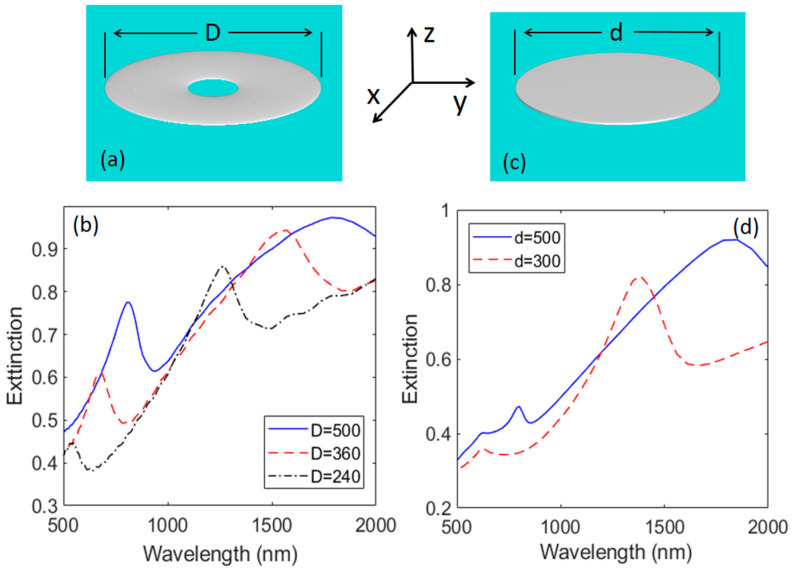
(**a**) Simulation structural model of an Ag N-donut with an external diameter of D. (**b**) Extinction of Ag N-donuts with D = 500 (solid line), 360 (dashed line), and 240 nm (dashed-dotted line). (**c**) Simulation structural model of an Ag N-discs with diameter d. (**d**) Extinction spectra of the Ag N-discs with d = 500 (solid line) and 300 nm (dashed line). The thicknesses of the N-donuts are considered to be 10 nm and those of N-discs to be 7 nm.

To further clarify the impact of structure, we considered Ag N-donuts with external diameters (D) of 500, 360, and 240 nm (Figure 7a). The thicknesses were considered to be about 10 nm based on the experiment. The results presented in Figure 7b show the plasmonic bi-resonance nature of such N-donuts. When the external diameter of the N-donuts is 500 nm, these resonances occur at 815 and 1833 nm (Figure 7b, solid line). As the diameter decreases both of these resonances are blue-shifted while they become more distinct (Figure 7b, dashed and dashed-dotted lines).

**Figure 8:**
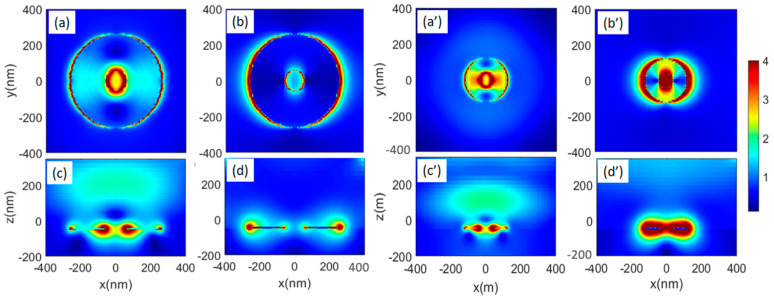
Mode field enhancement profiles associated with the N-donuts with D = 500 nm (**a**–**d**) and 240 nm (**a’**–**d’**). (**a**,**c**) are the profiles in the x–y and x–z planes at 814 and (**b**,**d**) are those at 1833 nm. (**a’**,**c’**) are profiles at 532 nm and (**b’**,**d’**) are those at 1270 nm.

The bi-resonance nature of N-donuts allows them to present a robust field enhancement over a wide range of wavelengths. To see this in Figure 8 we show the field enhancement profiles associated with the N-donuts with external diameters of 500 nm (a–d) and 240 nm (a’–d’). These results suggest that the shorter wavelength peaks seen in Figure 7b are associated with the inner openings of the N-donuts (inner modes). The field enhancement profiles of such modes in the x–y (Figure 8a) and x–z (Figure 8c) planes show high values around the inner edges of the N-donuts. As the wavelength increases, these modes are gradually transformed to the outer edge modes of the N-donuts (Figure 8b,d). The same scenario can also be seen for the case of N-donuts with an external diameter of 240 nm. As seen in Figure 8a’–d’ in this case the bi-resonance nature of plasmonic effects are more continuous, offering large plasmonic field enhancement over a wide range of wavelength. For both cases of D = 500 and 240 nm, around the wavelengths that the inner modes happen, the structures support some degree of optical field enhancement deep in the superstrate. For the near field, close to the surface of N-donuts, one expects a field enhancement factor of more than 4.

In the case of N-discs (Figure 7c), however, the prime feature of the plasmonic effect is associated with the dipolar nature of their edge modes. As seen in Figure 7d, in such a structure when the diameter is 500 or 300 nm, these modes happen at 1850 and 1385 nm, respectively. The weak peaks are seen at 800 and 620 nm could be associated with the weak cavity modes, generated by the relatively large lateral sizes of the N-discs. Figure S4 shows the mode field enhancement profiles in the x–y plane (Figure S4a,b) and the x–z plane (Figure S4c,d) when the diameter of the N-disc is 300 nm. Note that the field enhancement associated with cavity modes are rather weak. The dipolar-like mode is rather strong, but it happens in the infrared range.

## 4. Conclusions

In summary, we have developed a layer-by-layer growth process for the fabrication of MoS_2_ N-donuts/graphene nanohybrids SERS substrates. The MoS_2_ N-donuts growth process consists of two major steps: coating of (NH4)_2_MoS_4_ precursor at room temperature in air, followed by vapor transport anneal in sulfur vapor transfer process at 450 °C for 30 min. We have found that the predominant MoS_2_ N-donuts of the larger lateral dimension of 300–700 nm and height of 5–10 nm can be obtained by varying the precursor concentration to exceeding 0.20 wt% to promote nonuniform MoS_2_ conversion on larger precursor nuclei. This is in contrast to the formation of predominantly MoS_2_ N-discs of smaller lateral dimension at lower precursor concentrations down to 0.06 wt%. A SERS study using R6G probe molecules has shown that higher R6G SERS sensitivity has been observed on MoS_2_ N-donuts/graphene nanohybrid substrates than on the MoS_2_ N-discs/graphene ones. Specifically, the highest SERS sensitivity up to 2 × 10^−12^ M has been achieved on the MoS_2_ N-donuts/graphene nanohybrid SERS substrates made using 0.26 wt% precursor concentration. This is a half order of magnitude higher than 5 × 10^−12^ M on the MoS_2_ N-discs/graphene SERS substrates. FDTD simulation indicates that MoS_2_ N-donuts can better support LSPR than their MoS_2_ N-discs counterparts. This result illustrates the feasibility of tuning TMD nanostructure morphology for high-performance nonmetallic plasmonic nanohybrids for low-cost, large-scale applications in biosensing and other optoelectronics.

## Data Availability

Not applicable.

## Supplementary Materials

The following are available online at https://www.mdpi.com/article/10.3390/bios11120477/s1, Figure S1: (a) Raman spectra of pristine graphene (green, inset), and 0.10 wt% MoS_2_N-donuts/graphene (red), and 0.26 wt% MoS_2_N-discs/graphene (blue). (b) Raman spectrum of MoS_2_N- donuts (0.26 wt%) and discs (0.10 wt%)/graphene. All spectra were taken using 488 n, Figure S2: The enhanced Raman spectra of the R6G molecules 5 × 10^−5^M deposited on five MoS_2_/graphene nanohybrids substrates, MoS_2_ was synthesized with a precursor solution concentration of 0.06 wt % (a), 0.10 wt %, 0.20 wt %, 0.26 wt %, and 0.32 wt % (e), the spectra were collected from six batches of MoS_2_ NDs/graphene substrates samples, Figure S3: The intensities of the Raman at 613 cm^−1^ peak as a function of the R6G concentration on substrates using a linear scale (a, b) and a semi-logarithmic scale (c,d). The Raman excitation was at 532 nm, Figure S4: Mode field enhancement profiles in x–y planes of an N-disk with D = 300 nm at 620 nm (a) and 1385 (b). (c) and (d) show, respectively, their profiles in the x–z planes.
