# Supplementary material for: MoS2 Nanodonuts for High-Sensitivity Surface-Enhanced Raman Spectroscopy
Source: Biosensors (Basel). 2021 Nov 25;11(12):477. doi: 10.3390/bios11120477 (PMC8699280; doi:10.3390/bios11120477)
Supplement: Supplementary file 1 [file biosensors-11-00477-s001.zip › biosensors-1461797-supplementary.pdf]

biosensors

MDPI

Supporting Information

MoS_2 Nanodonuts for High-Sensitivity Surface-Enhanced Raman Spectroscopy

Samar Ali Ghopry [1,2,*], Seyed M. Sadeghi [3], Cindy L. Berrie [4] and Judy Z. Wu [1,*]

[1] Department of Physics and Astronomy, University of Kansas, Lawrence, KS 66045, USA
[2] Department of Physics, Jazan University, Jazan 45142, Saudi Arabia
[3] Department of Physics, The University of Alabama, Huntsville, AL 35899, USA; ss0013@uah.edu
[4] Department of Chemistry, The University of Kansas, Lawrence, KS 66045, USA; cberrie@ku.edu
* Correspondence: s461g593@ku.edu (S.A.G.); jwu@ku.edu (J.Z.W.)

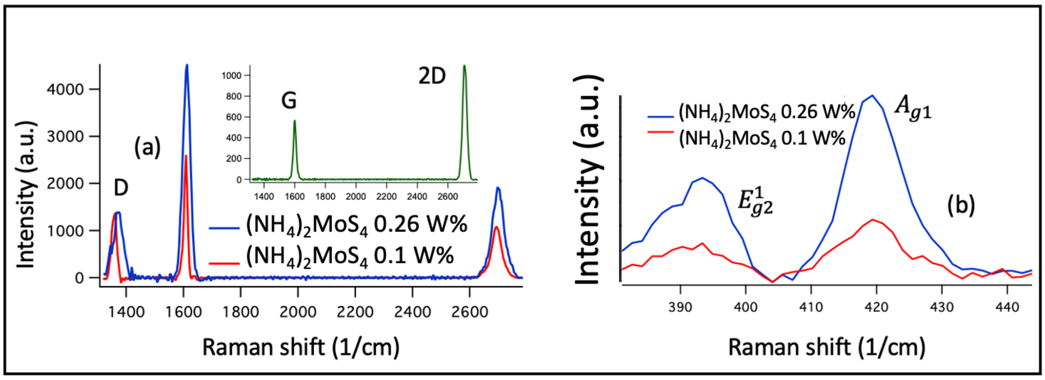

Figure S1. (a) Raman spectra of a pristine graphene (green, inset), and 0.1 wt% MoS_2N- donuts/graphene (red)., and 0.26 wt% MoS_2N-discs/graphene (blue). (b) Raman spectrum of MoS_2N- donuts (0.26 wt%) and discs (0.1 wt%)/graphene. All spectra were taken using 488 nm.

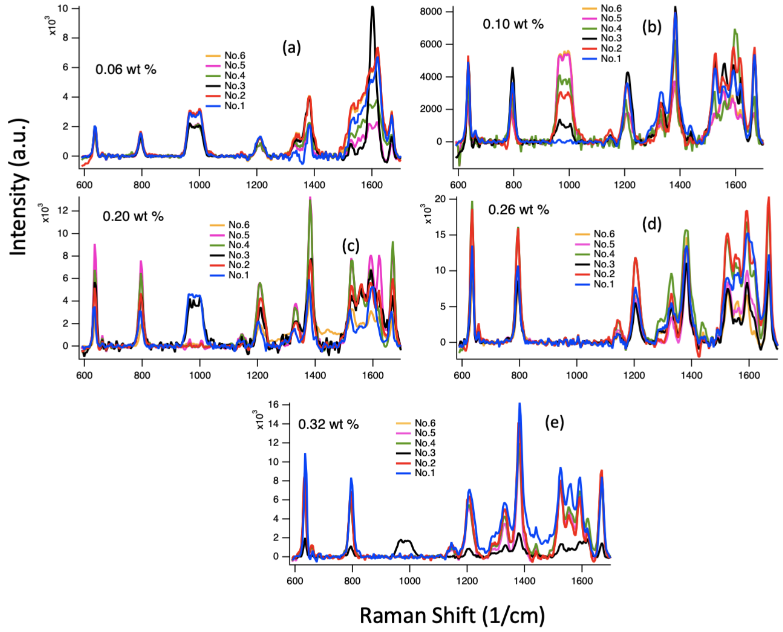

Figure S2. The enhanced Raman spectra of the R6G molecules 5×10^{-5}M deposited on five MoS_2/graphene nano-hybrids substrates, MoS_2 was synthesized with precursor solution concentration of 0.06 wt % (a), 0.10 wt %t, 0.20 wt %, 0.26 wt % and 0.32 wt % (e), the spectra were collected from six batches of MoS_2 NDs/graphene substrates samples.

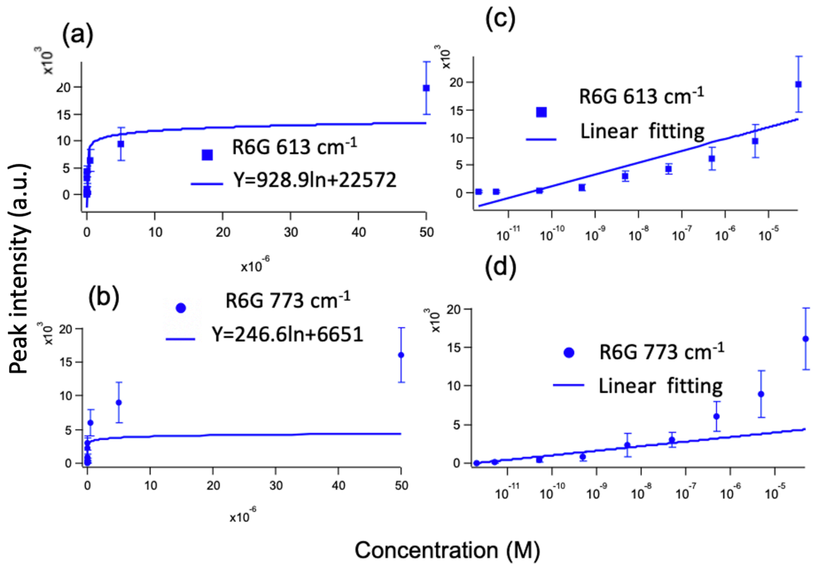

Figure S3. The intensities of the Raman at 613 cm^{-1} peak as a function of the R6G concentration on substrates using a linear scale (a, b) and a semi-logarithmic scale (c, d). The Raman excitation was at 532 nm.

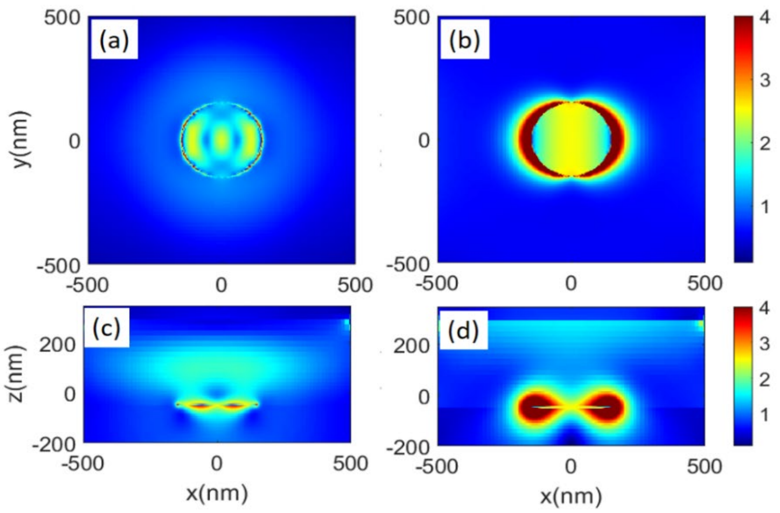

Figure S4. Mode field enhancement profiles in x-y planes of a N-disk with D=300 nm at 620 nm (a) and 1385 (b). (c) and (d) show, respectively, their profiles in the x-z planes.
